# Leukaemic cells expressing ETV6::FRK are sensitive to dasatinib in vivo

**DOI:** 10.1002/jha2.701

**Published:** 2023-05-02

**Authors:** Azusa Mayumi, Toshihiko Imamura, Hideki Yoshida, Shinya Osone, Takahiko Yasuda, Tomoko Iehara

**Affiliations:** ^1^ Department of Paediatrics Kyoto Prefectural University of Medicine Kyoto Japan; ^2^ Clinical Research Center Nagoya Medical Centre National Hospital Organization Nagoya Japan

**Keywords:** acute lymphoblastic leukaemia, B‐ALL, dasatinib, ETV6::FRK

## Abstract

ETV6::Fyn‐related kinase (FRK), which is a Src family tyrosine‐kinase‐related fusion gene and firstly identified in our patient with paediatric high risk B cell precursor acute lymphoblastic leukaemia (B‐ALL), has no evidence of efficacy of tyrosine kinase inhibitor in vivo. We performed functional analysis of ETV6::FRK to establish molecular targeting therapy and determined that dasatinib could abrogate proliferation activity of ETV6::FRK through the repression of FRK‐STAT3/STAT5 pathway in vitro and significantly extended the survival time of the xenografted mice in vivo (*p* < 0.01). Our data support the potential of dasatinib as a therapeutic option for patients with B‐ALL harboring FRK rearrangements.

## 1 INTRODUCTION

Fyn‐related kinase (FRK) is a member of the breast tumuor kinase family of tyrosine kinases, which are related to Src family tyrosine kinases. FRK was originally attributed a tumour‐suppressive function; however, further functional characterization in diverse cancer types has revealed that it can also play oncogenic roles [1]. ETV6::FRK, which is chimeric fusion gene comprising the oncogenic transcription factor, *ETV6* (ETS variant transcription factor 6, also known as *TEL*), and FRK, was first identified in acute myeloid leukaemia (AML) [2]. Expression of ETV6::FRK induces IL‐3‐independent growth in Ba/F3 cells, suggesting that this fusion is a critical pathogenic driver of leukaemogenesis [2]. Other FRK‐related fusion genes, including *CAPRIN1*::FRK, *PABPC1*::FRK, and *MAPK9*::FRK, have been identified in ALK‐negative anaplastic large cell lymphoma (ALCL) [3]. These findings suggest that FRK‐related fusion genes are recurrent in haematological malignancies and critical drivers of leukaemogenesis. As FRK is an ABL‐class kinase of the Src family, the tyrosine kinase inhibitor, dasatinib, has been proposed as a potentially effective treatment for targeting cells harboring FRK‐related fusion genes; however, its efficacy has only been reported in vitro against cells carrying *CAPRIN1*::FRK [3]. Thus, targeting FRK‐related fusion proteins with dasatinib warrants more extensive investigation. Herein, we first identified the ETV6::FRK fusion gene in a patient with high‐risk B‐cell precursor acute lymphoblastic leukaemia (B‐ALL) and performed functional analysis of cells expressing ETV6::FRK to evaluate the efficacy of targeting FRK with dasatinib in vitro and in vivo.

### 1.1 Patient and methods

**FIGURE 1:**
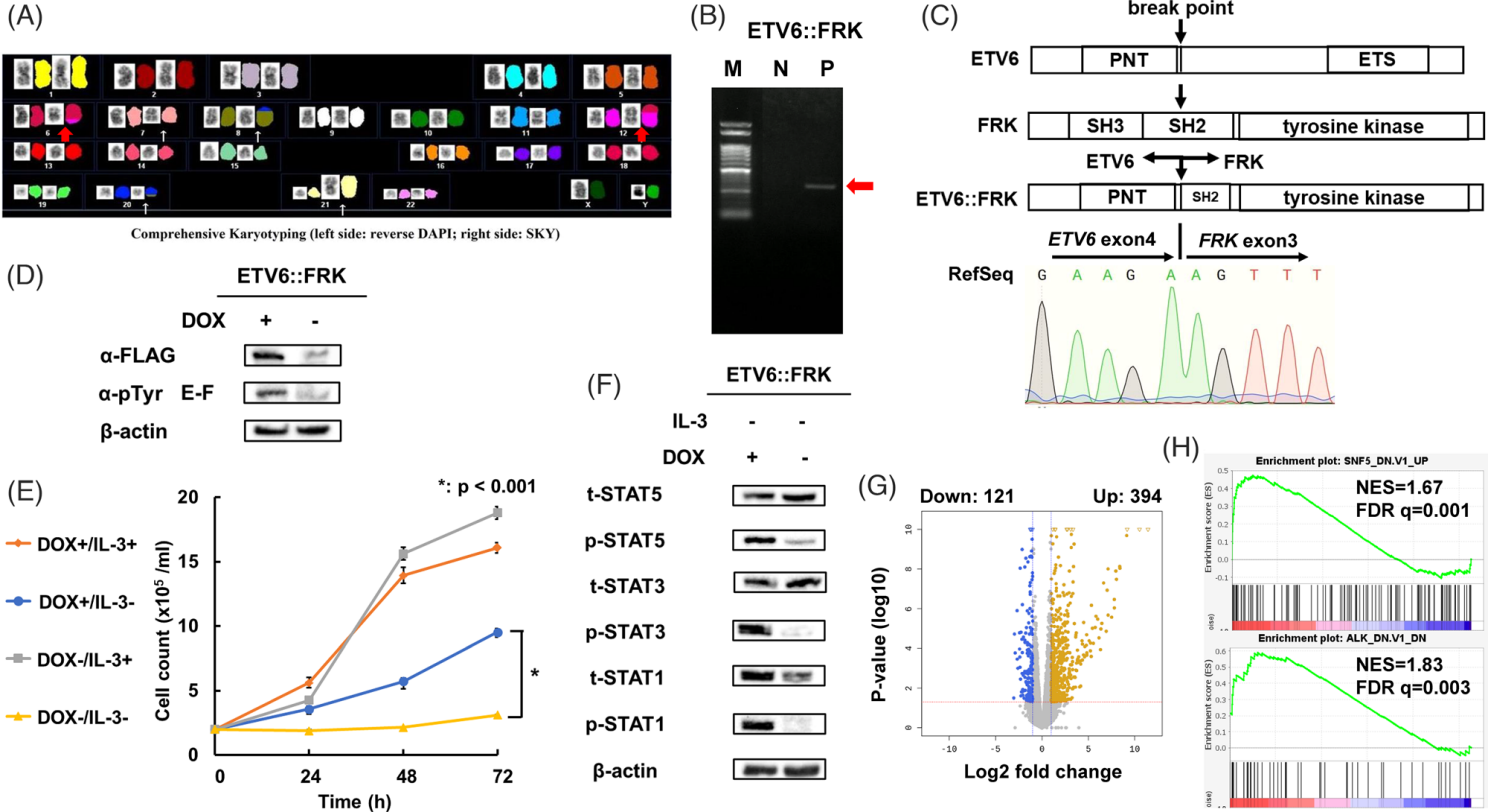
Identification of ETV6:: Fyn‐related kinase (FRK), and analysis of Ba/F3‐ETV6::FRK cell proliferation and gene expression. (A) Comprehensive karyotype analysis of peripheral blood cells from our patient by a combination of 4,6‐diamidino‐2‐phenylindole (DAPI) banding and spectral karyotyping (SKY) showing 46,XY,t(6;12)(q21;p13). (B) RT‐PCR analysis using primers in *ETV6* exon 4 and FRK exon 4 in patient and control samples amplified an ETV6::FRK junction DNA fragment from patient, but not control, cDNA samples. N, negative control; P, patient (325 bp); M, 100 bp DNA ladder. (C) Sanger sequencing of the PCR product confirmed an in‐frame fusion of FRK exon 3 to *ETV6* exon 4. Schematics of the structures of ETV6, FRK, and ETV6::FRK (fusion) proteins. Arrows indicate the locations of the t(6;12)(p21;q13) fusion points. (D) Doxycycline (DOX)‐induced protein expression of FLAG‐tagged ETV6::FRK and phosphorylated tyrosine were detected by western blot analysis, using anti‐FLAG and anti‐phosphotyrosine antibodies. Representative images from three independent experiments are shown. (E) Ba/F3 cells expressing ETV6::FRK (DOX+) proliferate in the absence of interleukin (IL)‐3. Results are presented as mean ± standard deviation of data obtained from triplicate experiments (DOX+/IL‐3‐ vs. DOX‐/IL‐3+; *p < 0.001). (F) Western blots demonstrating that STAT5, STAT3 and STAT1 are constitutively phosphorylated in Ba/F3 cells expressing ETV6::FRK (DOX+). Whole cell lysates of Ba/F3 cells expressing ETV6::FRK (DOX+ or DOX–) were transferred and immunoblotted with the indicated antibodies. Representative images from three independent experiments are shown. (G) Volcano plot illustrating up‐ and down‐regulated genes, with log_2_ fold change >2.0 (yellow, 394 genes) or <–2.0 (blue, 121 genes), respectively, with adjusted *p*‐values (‐log 10), differentially expressed in Ba/F3‐ETV6::FRK (DOX+) compared with non‐induced (DOX‐) cells. (H) Gene set enrichment analysis (GSEA) comparison of Ba/F3‐ETV6::FRK (DOX+) versus non‐induced (DOX‐) cells, showing enrichment of SNF5 and ALK target genes. DOX, doxycycline; E‐F, ETV6::FRK; FDR, false discovery rate; PNT, pointed domain; ETS, erythroblast transformation‐specific domain; SH3, Src homology 3 domain; SH2, Src homology 2 domain; Tyr, tyrosine; NES, normalised enrichment score.

Our patient was 11‐year‐old boy with B‐ALL who was resistant to induction chemotherapy. Analysis of leukaemic blasts by spectral karyotyping fluorescence in situ hybridization revealed a 46,XY,t(6;12)(q21;p13) karyotype (Figure 1A). To search for the potential fusion gene, target capture RNA‐Seq was performed using RNA isolated from the diagnostic leukaemic sample, as described previously [4]. Chimera gene‐inducible cell lines were generated by retroviral transfection using the Retro‐X Tet‐On Advanced Inducible Expression System (Takara Bio, USA). Generation of retroviral supernatants by Plat‐E cells, infection and transduction of the interleukin (IL)−3‐dependent murine pro‐B‐cell line, Ba/F3 cells, was carried out as previously described [5]. Cell proliferation assay, cytotoxic assay and apoptosis assay were also performed as described previously [5]. To characterize the transcriptome profile of established cells, whole transcriptome sequencing (WTA) was conducted (Macrogen Japan, Tokyo, Japan). Gene set enrichment analysis (GSEA) was performed using GSEA software (v4.2.2; Broad Institute, Cambridge, MA, USA) with oncogenic gene sets (C6) from the GSEA Molecular Signatures Database [6]. Gene sets with nominal *p*‐values < 0.05 and FDR *q*‐values < 0.25 were considered significantly different. To establish xenograft model, NOD.Cg‐*Prkdc^scid^Il2rg^tm1Wjl^*/SzJ (NSG) mice were used (Jackson Laboratory, Yokohama, Japan). Transplanted cell burden was monitored using an IVIS Lumina Series III system (PerkinElmer, Waltham, MA, USA), as described previously [7]. All mouse studies were approved by institutional committees. An unpaired two‐tailed Student's *t*‐test was used to determine the significance of differences and *p*‐values < 0.05 considered statistically significant. Kaplan–Meier curves for survival analyses were compared using the log‐rank test.

## 2 RESULTS AND DISCUSSIONS

Target capture mRNA sequencing revealed an in‐frame fusion of *ETV6* (NM_001987) exon 4 to FRK (NM_002031) exon 3, generating an ETV6::FRK fusion transcript. RT‐PCR followed by Sanger sequencing of the PCR product confirmed the presence of ETV6::FRK (Figure 1B, C). The fusion point of the chimeric gene was the same as that previously reported in AML [2].

The ETV6::FRK fusion gene encodes a chimeric protein in which the entire pointed (PNT) oligomerization domain of ETV6 and the kinase domain of FRK are retained (Figure 1C). ETV6::FRK disrupts the Src homology 3 (SH3) domain and Src homology 2 (SH2) domains of FRK, which are required to maintain Src family kinases in an inactive state, which would be predicted to result in constitutive activation of the ETV6::FRK tyrosine kinase, relative to wild‐type FRK. Moreover, retention of the PNT oligomerization domain of ETV6 in ETV6::FRK interferes with ETV6 transcriptional repression activity by facilitating heterodimerization with wild‐type ETV6 [2].

To investigate the functional consequences of the ETV6::FRK fusion protein, we generated a construct expressing ETV6::FRK and introduced it into Ba/F3 under a tetracycline‐inducible gene expression system, to generate ETV6::FRK‐expressing cells (Ba/F3‐ETV6::FRK). Induction of ETV6::FRK expression by administration of doxycycline was confirmed by western blot analysis (Figure 1D). As the FRK kinase domain was present in ETV6::FRK (Figure 1C), we analysed whether the fusion protein itself was constitutively tyrosine phosphorylated. Western blotting analysis showed that Ba/F3‐ETV6::FRK exhibited constitutive phosphorylation of tyrosine residue of ETV6::FRK (Figure 1D). ETV6::FRK transformed Ba/F3 cells toward IL‐3‐independent proliferation, implicating a role in oncogenic transformation, as described previously [2] (Figure 1E). The STAT3 pathway is downstream of FRK kinase [1]; therefore, we next examined whether ETV6::FRK activated downstream STAT proteins. As shown by western blotting analysis, Ba/F3‐ETV6::FRK expression led to constitutive phosphorylation of STAT1, STAT3 and STAT5 (Figure 1F).

Next, to investigate which pathways were affected by ETV6::FRK, we compared gene expression profiles from Ba/F3‐ETV6::FRK cells with those of non‐DOX‐induced control cells using WTA and GSEA. WTA revealed that, among 14238 genes analysed, expression of 394 and 121 were increased and decreased (log_2_ fold change ≥ |2.0|; adjusted *p* < 0.05), respectively, in Ba/F3‐ETV6::FRK cells compared with controls (Figure 1G). The top 20 up‐ and down‐regulated genes are listed in Table S1. GSEA of oncogenic gene sets (C6) from the GSEA Molecular Signatures Database [6] revealed that, compared with control cells, Ba/F3‐ETV6::FRK cells were enriched for up‐regulation of SNF5 target genes and down‐regulation of RB target genes involved in cell cycle regulation (Figure 1H, Table S2). Ba/F3‐ETV6::FRK cells were also enriched for down‐regulation of ALK target genes, consistent with previous reports that FRK and ALK regulatory networks share several proteins in common, including STAT3 [1, 8]. Further, the observed enrichment for up‐regulation of EGFR target genes may reflect that the EGFR signalling inhibition function of FRK is diminished in the context of ETV6::FRK, relative to wild‐type FRK [9].

**FIGURE 2:**
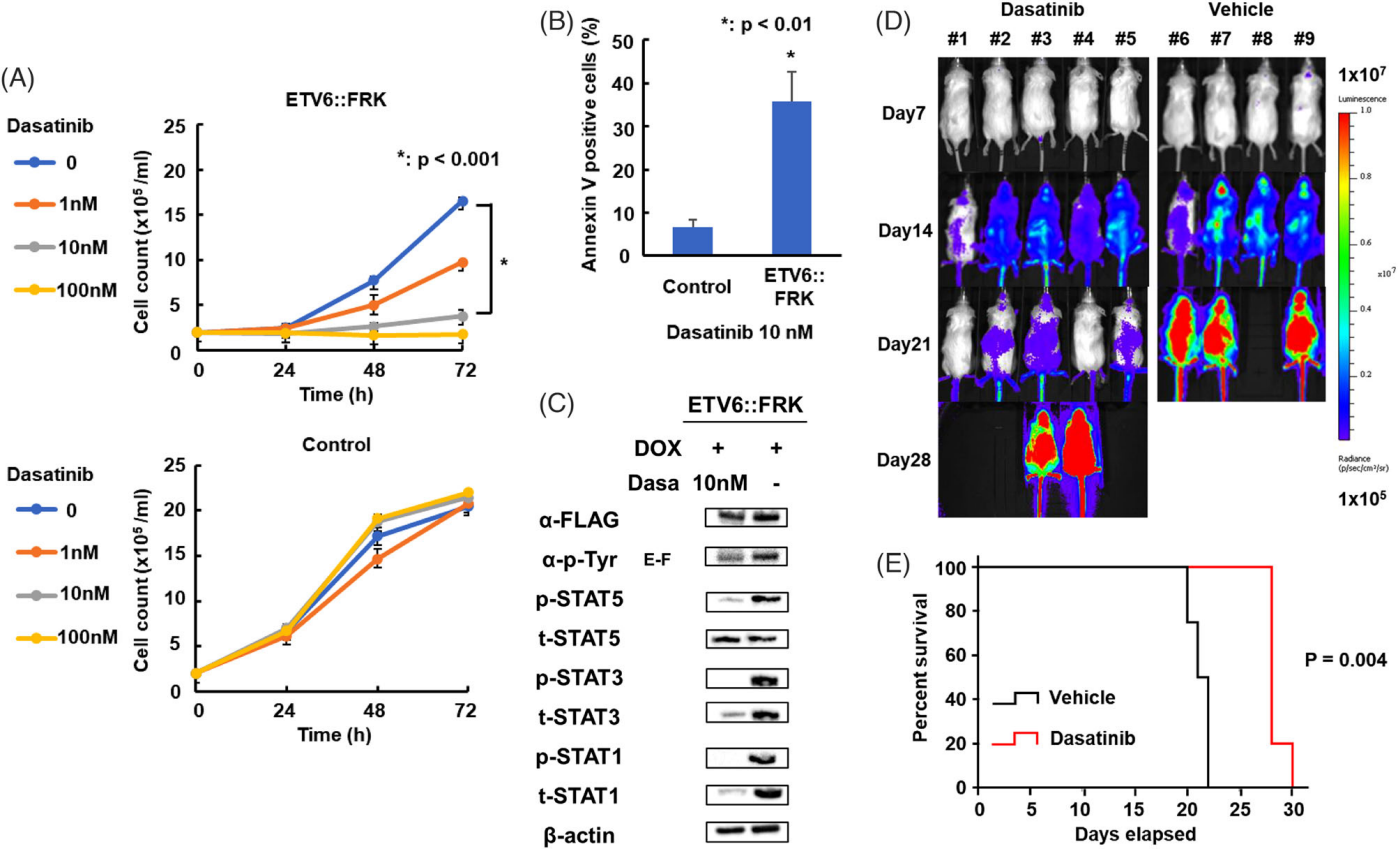
Effects of dasatinib on ETV6:: Fyn‐related kinase (FRK) in vitro and in vivo. (A) ETV6::FRK‐expressing Ba/F3 cells, cultured in the absence of interleukin (IL)‐3, and control non‐induced (DOX‐) cells were treated with the indicated concentrations of dasatinib, and cell survival was quantified after 24, 48 and 72 h. Ba/F3‐ETV6::FRK cell proliferation was inhibited in a dose‐dependent manner by treatment with dasatinib. Data are presented as mean ± standard deviation from three independent measurements (DOX+/IL‐3‐ vs. DOX+/IL‐3‐ with dasatinib 10 nM; *p < 0.001). (B) IL‐3‐independent Ba/F3 cells expressing ETV6::FRK were treated with dasatinib (10 nM, 48 h), and then apoptotic cells were analysed by annexin V/propidium iodide (PI) staining and flow cytometry. Early apoptotic cells were annexin V‐positive; late apoptotic cells were annexin V‐ and PI‐positive. Mean ± standard deviation values of three independent replicates are shown. *p < 0.01. (C) Western blot analysis showing the effect of dasatinib treatment on Ba/F3‐ETV6::FRK cells grown in the absence of IL‐3. ETV6::FRK phosphorylated tyrosine and phosphorylation of STAT5, STAT3 and STAT1 were reduced by dasatinib (10 nM). Representative images from three independent experiments are shown. (D) Bioluminescent images of tumour growth over time in individual mice (dasatinib, *n* = 5; vehicle, *n* = 4). (E) Kaplan–Meier plots of overall survival (dasatinib, *n* = 5; vehicle, *n* = 4). The dasatinib‐treated group achieved prolonged tumour control compared with the vehicle control group. Log‐rank test: *p* < 0.01. Dasa, dasatinib; DOX, doxycycline; E‐F, ETV6::FRK; Tyr, tyrosine.

To investigate whether dasatinib can block constitutive phosphorylation of ETV6::FRK and inhibit IL‐3‐independent proliferation of Ba/F3‐ETV6::FRK, we treated IL‐3‐independent Ba/F3‐ETV6::FRK with dasatinib (1, 10, or 100 nM), and assessed the effect on proliferation and viability. Cytotoxicity assays revealed that dasatinib suppressed Ba/F3‐ETV6::FRK cell proliferation in a dose‐dependent manner (IC50, 1.64 ± 0.02 nM) (Figure 2A). Annexin V/PI staining demonstrated that around 35% of Ba/F3‐ETV6::FRK cells treated with dasatinib (10 nM, 48 h) were apoptotic (Figure 2B), while dasatinib had no effect on control cell viability (35% vs. 7%; *p* < 0.01). Western blot analysis showed decreased FRK, STAT1, STAT3 and STAT5 phosphorylation on treatment with 10 nM dasatinib (Figure 2C). These findings suggest that dasatinib selectively inhibits proliferation induced by ETV6::FRK.

To verify the effects of dasatinib in vivo, we established xenograft models using Ba/F3‐ETV6::FRK cells expressing firefly luciferase (Ba/F3‐ETV6::FRK‐FFLuc). Ba/F3‐ETV6::FRK‐FFLuc cells were injected intravenously into NSG mice, and disease progression was then monitored by assessing Ba/F3‐ETV6::FRK‐FFLuc burden, revealing that dasatinib suppressed Ba/F3‐ETV6::FRK‐FFLuc cell proliferation (Figure 2D). Further, survival time was significantly prolonged in the dasatinib‐treated group (*p* = 0.004; Figure 2E). These findings suggest that dasatinib treatment suppressed leukaemic cell proliferation and prolonged xenograft mouse survival in vivo. In the vehicle control group, one mouse died just after intravenous injection of Ba/F3‐ETV6::FRK‐FFLuc cells into the tail vein on day 0. The cause of death may have been pulmonary embolism, and the data from this mouse were excluded from subsequent analyses.

In this study, we analysed the functional consequences of the ETV6::FRK fusion, which we first identified in a patient with B‐ALL. The ETV6::FRK chimeric protein enhanced cell proliferation and constitutional tyrosine autophosphorylation of ETV6::FRK was critical for this proliferative activity. Although the downstream signalling pathways mediated by ETV6::FRK were not fully elucidated in previous studies [2], our findings reveal that ETV6::FRK is catalytically active in the FRK‐STAT pathway, as evidenced by the observation of constitutive phosphorylation of STAT3, STAT1 and STAT5. These data are consistent with another report that the FRK fusion gene, *CAPRIN1*::FRK, identified in ALCL, induced phosphorylation of STAT3 in the Ba/F3 model [3].

Dasatinib is considered potentially effective for the treatment of cancers with FRK rearrangements; however, evidence of dasatinib use is sparse and limited to in vitro studies; preclinical data showed that dasatinib inhibits CAPRIN1::FRK‐driven cell growth in vitro in ALK‐negative ALCL [3]. Here, we clearly demonstrate that dasatinib is effective in inhibiting ETV6::FRK‐driven cell growth in vitro and in vivo, as demonstrated using xenograft models. Our data support the hypothesis that dasatinib is effective for treatment of haematological malignancies harboring FRK‐related fusion genes.

In summary, we report: (1) the first identification of the rare kinase‐related fusion gene, ETV6::FRK, in a patient with B‐ALL; (2) that ETV6::FRK induces constitutive kinase activity, STAT activation, and cell proliferation; and (3) that dasatinib shows cytotoxic effects against Ba/F3‐ETV6::FRK cells in vitro and significantly prolongs the survival of mice xenografted with Ba/F3‐ETV6::FRK cells in vivo.

## AUTHOR CONTRIBUTIONS

AM and TI designed the research, performed the experiments, analysed the data, generated the figures and wrote the manuscript. TY performed targeted capture mRNA sequencing. HY, SO and TI supervised the research and participated in editing the manuscript. All authors approved the final version of the manuscript.

## CONFLICT OF INTEREST STATEMENT

The authors declare no competing financial interests.

## ETHICS STATEMENT

Written informed consent was obtained from the parents of the patient, and informed assent was obtained from the patient.

## Supporting information

Supplementary information

Supplementary information

Supplementary information

## Data Availability

Original data presented in this manuscript are available upon request addressed to the corresponding author.
