# Supplementary material for: Leukaemic cells expressing ETV6::FRK are sensitive to dasatinib in vivo
Source: EJHaem. 2023 May 2;4(3):751–5. doi: 10.1002/jha2.701 (PMC10435712; doi:10.1002/jha2.701)
Supplement: Supplementary file 1 — Supplementary information [file JHA2-4-751-s001.docx]

**Supplemental Table Legends**

**Supplemental Table 1. Up- and down-regulated genes in Ba/F3-ETV6::FRK cells.** The top 20 up- and down-regulated genes in Ba/F3-ETV6::FRK cells. Top 20 genes showing higher or lower gene expression levels in Ba/F3-ETV6::FRK (DOX-on.) cells, defined as a > log2-fold difference in expression level relative to non-induced (DOX-off.) cells, determined by transcriptome analysis. DOX, doxycycline; FDR, false discovery rate.

**Supplemental Table 2.** **Enriched gene sets in Ba/F3-ETV6::FRK cells.** All enriched gene sets identified by GSEA comparison of Ba/F3-ETV6::FRK (DOX+) and non-induced (DOX-) cells. Significant gene sets were selected according to a difference in nominal p-value of < 0.01 and FDR q-value of < 0.25. NES, normalised enrichment score; FDR, false discovery rate; NOM, nominal.
